# Service Delivery Considerations for Introducing New Injectable Contraceptives Lasting 4 and 6 Months in Nigeria and Uganda: A Qualitative Study

**DOI:** 10.9745/GHSP-D-23-00214

**Published:** 2023-12-22

**Authors:** Rebecca L. Callahan, Holly M. Burke, Anna Lawton, Funmilola M. OlaOlorun, Fredrick Mubiru, Helen Anyasi, Christina M. Wong, Dieudonné Bidashimwa, Marissa Velarde, Lucy W. Ruderman

**Affiliations:** a Product Development and Introduction, FHI 360, Durham, NC, USA.; b Reproductive, Maternal, Newborn, Child Health, FHI 360, Durham, NC, USA.; c College of Medicine, University of Ibadan, Ibadan, Nigeria.; d FHI 360, Kampala, Uganda.; e FHI 360, Abuja, Nigeria.; f Global Health and Population Research, FHI 360, Durham, NC, USA.

## Abstract

**Background:**

New family planning (FP) product introduction requires understanding the target market and support from stakeholders from across the health sector. We aimed to understand the perspectives of FP providers and other stakeholders on the potential introduction of new subcutaneous (SC) depot medroxyprogesterone acetate (DMPA) injectable contraceptives lasting 4 and 6 months in Nigeria and Uganda.

**Methods:**

Between July 2021 and February 2022, we conducted 48 in-depth interviews (IDIs) and 11 focus group discussions (FGDs) with FP providers and other stakeholders involved with service delivery, program management, and policymaking in Lagos and Abuja in Nigeria and Kampala and Luwero in Uganda. IDIs and FGDs explored respondents' reactions to and preferences for the new injectables lasting 4 and 6 months.

**Results:**

Most respondents liked the idea of longer-acting DMPA-SC products, noting the potential for reduced facility visits for clients and workloads for providers, cost savings for users and the health system, and potential for improved commodity logistics. Some nonproviders raised concerns about confusion among providers and clients with the availability of multiple injectable products; however, providers did not share this concern. The greatest interest among all groups was for the 6-month injectable, even without the option for self-injection. Several respondents reported that self-injection is not widespread in either context, and some noted that contact with a provider would be important for products with longer durations. Respondents' acceptability of the new injectables assumed that side effects would be no worse than the existing 3-month product.

**Conclusions:**

Family planning stakeholders in Nigeria and Uganda are supportive of expanding the method mix with new injectables, which they see as having the potential to meet the needs of more users. Concerted engagement of health providers, policymakers, and the community will be necessary for successful introduction once these new contraceptive products are available.

## Abstract (precis)

Family planning stakeholders in Nigeria and Uganda are interested in expanding the range of injectable contraceptives offered in their countries, and their engagement will be key to ensuring the successful introduction and scale-up of these new products.

## Teaser

**Key Findings:**

- Family planning (FP) stakeholders, including providers, policymakers, and program implementers, are interested in the possibility of new injectables lasting 4 and 6 months for expanding the method mix and broadening contraceptive choice.
- FP stakeholders are most excited at the prospect of a 6-month injectable coming to market.
- FP stakeholders do not view self-injection as a key requirement for new injectable methods.

**Key Implications:**

- This research provides insight into specific opportunities and concerns that stakeholders from across the FP community in Nigeria and Uganda identify for the introduction of new contraceptive injectables.

See related article by Burke et al.

## BACKGROUND

Despite substantial gains in access to and use of contraception over the last 50 years, many women and couples continue to have an unmet need for family planning (FP) and experience unintended pregnancy. In many sub-Saharan African countries, contraceptive prevalence rates have plateaued, with access to contraception remaining constrained for many rural, poor, and vulnerable populations.^1^^–^^3^ The development of new contraceptive methods that better meet users' needs, particularly those in resource-constrained and remote locations, holds the promise of increasing method use and satisfaction. Studies have shown that expanding the method mix and improving features of current methods can lead to increased contraceptive use.^4^

Injectables are a large proportion of the method mix across sub-Saharan Africa, and many countries in the region have recently expanded access to the subcutaneous formulation of depot medroxyprogesterone acetate (DMPA-SC), known by the brand name Sayana Press (Pfizer, Inc.). In Uganda, injectables make up 43% of the method mix, with 18% of use being DMPA-SC. In Nigeria, 17% of contraceptive users use injectables, with a growing proportion (1%) using DMPA-SC.^5^^,^^6^ Packaged in the single-use Uniject injection system, DMPA-SC may be injected either by users or providers. Self-injection of DMPA-SC has gained in popularity among women for its convenience, safety, and effectiveness^7^ and is recommended by the World Health Organization in their self-care guidelines.^8^

DMPA-SC is currently labeled for 3 months of use.^9^ However, recently generated evidence shows that the standard DMPA-SC formulation is effective for a longer period.^10^^,^^11^ In a multicountry clinical trial of 750 women, no one who reinjected with DMPA-SC every 4 months became pregnant, and all study participants maintained effective levels of the contraceptive hormone medroxyprogesterone acetate over the 4-month reinjection interval.^12^

Another multicountry clinical trial was recently launched that will assess the safety, efficacy, and acceptability of a potential 6-month injectable contraceptive [ISRCTN #62695528]. However, unlike existing DMPA-SC, this product would be in traditional vial and syringe presentation and administered subcutaneously by a health provider.

Although the clinical data indicate high efficacy of existing DMPA-SC for 4 months, the path to introduction for a 4-month injectable—either through a change in the existing product labeling, development of a dedicated product, or some other route—could be lengthy and complicated. Understanding the potential interest in such a product among users, providers, and other stakeholders is key for donors and product developers considering investment in the method. Similarly, understanding the market for a new 6-month injectable will be necessary if and when that product shows efficacy. Introduction of these new injectables will require considerable input and buy-in from stakeholders at all levels of local and national health systems, as well as the broader global FP community.

Understanding the potential interest in new contraceptive products among users, providers, and other stakeholders is key for donors and product developers considering investment in the methods.

Stakeholder engagement is critical for the launch of any new contraceptive innovation, as evidenced by the recent successful introduction of DMPA-SC in several sub-Saharan African countries.^13^ During pilot introductions in Burkina Faso, Niger, Senegal, and Uganda, country stakeholders shared their perspectives on key questions, including the number of doses, intended populations of users (first-time mothers, young and adolescent women), the coexistence of DMPA-SC with intramuscular DMPA (DMPA-IM) and other methods on the market, and the potential effects of community-based distribution approaches. Stakeholders' opinions on these questions led to the formation of a global monitoring system that provided relevant information for decisions around investments in and scale-up of DMPA-SC in those countries.^14^ In Uganda, stakeholders identified DMPA-SC's introduction as an opportunity to improve general FP access, including strengthening supply and service quality and thus better meeting clients' needs.^15^ Engagement with local stakeholders was also instrumental in generating demand for DMPA-SC.^15^

This article describes findings from research conducted with key FP stakeholders in Uganda and Nigeria on the potential introduction of a 4-month DMPA-SC as well as a potential 6-month product currently under clinical development. The research aimed to identify potential opportunities and challenges for the introduction of these new injectable products in contexts where 3-month DMPA-SC is in varying stages of scale-up. More specifically, the research aimed to explore FP stakeholder views on how these potential new injectables would fit into the existing method mix, what additional information would be needed for introduction, how they thought they would be accepted by users, and their views on specific product characteristics. This study is part of a broader market research activity that assessed potential interest in contraceptive injectables of multiple durations among both FP stakeholders and users. Results of the user-focused research are described elsewhere.^16^

## METHODS

### Study Setting

We conducted this qualitative study in Lagos and Abuja, Nigeria, and Kampala and Luwero, Uganda. Although DMPA-IM is the most commonly used injectable in both settings, DMPA-SC use has grown in recent years, bolstered by dedicated introduction efforts. For example, both countries have a DMPA-SC Working Group of national stakeholders that meets regularly to discuss product scale-up. Uganda and Nigeria are also priority countries for the Children's Investment Fund Foundation under their Delivering Innovation in Self Care grant, which seeks to generate substantial market growth and demand generation for self-injection through consumer marketing.^17^

We chose Kampala and Abuja for this research because they are capital cities where national-level stakeholders reside and work. In Lagos, the commercial capital of Nigeria, other key stakeholders reside and an active network of community health workers is engaged in FP service delivery in peri-urban areas of the state. In Uganda, Luwero was selected as an accessible rural district from which to solicit service delivery perspectives outside of the capital.

DMPA-SC was approved and registered by Nigeria's National Agency for Food and Drug Administration and Control in 2011 and introduced with the brand name Sayana Press to the private sector in 2015 by the social marketing enterprise, DKT. Introduction to the public sector commenced in 2016, coordinated by the United Nations Population Fund, and implemented by 3 nongovernmental organizations (NGOs): Action Health Incorporated, Association for Reproductive & Family Health, and the Planned Parenthood Federation of Nigeria. DMPA-SC's regulatory approval was updated in 2016 to include self-injection and scale-up accelerated with the implementation of the *National DMPA-SC Accelerated Introduction and Scale-up Plan 2018-2022*, followed by *National Guidelines for the Introduction and Scale-up of DMPA-SC Self-injection* released in 2019. Other activities supporting expansion of DMPA-SC in Nigeria include revision of the national task-shifting/task-sharing policy to allow more cadres of health workers to provide DMPA-SC, inclusion of DMPA-SC on the essential and approved patent medicines lists, and inclusion in the country's FP costed implementation plan for 2019–2023.^18^^,^^19^

Uganda's first experience with DMPA-SC was during an acceptability study conducted between 2012 and 2013 by FHI 360 in partnership with the Ministry of Health (MOH).^20^^,^^21^ Uganda established introduction policies for DMPA-SC in 2014, and over the next 2 years, DMPA-SC was piloted through community health workers.^14^^,^^22^ After the successful pilot, DMPA-SC was added to Uganda's essential medicines list and clinical guidelines in 2016, and the DMPA-SC Scale-Up Task Force was established. This task force consists of nearly 20 organizations and has been key to ensuring ownership and coordination of DMPA-SC and DMPA-SC self-injection introduction and scale-up as part of the FP method mix.^22^ A DMPA-SC self-injection study was conducted in 2016–2017, which led to DMPA-SC being registered for self-injection in 2017 and the policy authorization for self-injection of DMPA-SC in 2020.^22^^,^^23^

### Study Population and Sampling

We purposefully selected respondents for this study based on their involvement with FP service delivery, program management, and policymaking, including involvement in the introduction and/or scale-up of DMPA-SC in Uganda and Nigeria. In each country, we aimed to conduct up to 25 in-depth interviews (IDIs) with FP stakeholders, up to 5 mini-focus group discussions (FGDs) (dyads or triads) with health care providers, including community health workers, as well as an FGD with up to 10 DMPA-SC working group members for a total of up to 50 participants per country. These sample sizes were based on evidence that 3 to 6 FGDs per subpopulation and up to 8 to 12 IDIs would yield 80% of relevant themes on a focused topic, including the most salient themes.^24^^,^^25^ We aimed to conduct more IDIs and FGDs to ensure we had sufficient sample size to reach thematic saturation.

For mini-FGDs, country-based site investigators (authors FMO and FM) identified health facilities in Luwero, Uganda, and Lagos, Nigeria, that currently offer DMPA-SC and invited public sector FP providers, including clinic-based midwives and nurses as well as community health workers, to participate. In Lagos, a request was made to the state reproductive health coordinator, who identified and invited the public sector nurses and midwives from 2 different facilities. In Luwero, the assistant district health officer in charge of FP and maternal health helped identify providers at 2 public health facilities. For IDIs, the site investigators compiled lists of stakeholders in the public and private sectors. In Lagos, 2 implementing partners involved in the training of private sector staff to administer injectables provided lists of trained providers. Both lists contained geographic information, and individuals were randomly selected to ensure wide representation across the state. In Luwero, private sector providers were identified using contact information collected as part of prior FHI 360 work and verified with the district health office to confirm that they were still in service. Finally, up to 10 members of the DMPA-SC Working Group in each country were invited to participate in IDIs or FGDs. DMPA-SC Working Group members who participated in FGDs were ineligible to participate in an IDI. All participants needed to be at least age 18 years and willing to be audio-recorded to participate. Additionally, providers must have administered DMPA-SC to at least 1 client in the past 3 months.

### Data Collection

The site investigators conducted all IDIs and FGDs. Both are experienced FP researchers in their respective countries. They knew some but not all nonprovider stakeholders from professional contexts before this study, but they did not have any relationship with the FP providers before the study. Stakeholder IDIs and mini-FGDs with providers and DMPA-SC working group members followed semistructured discussion guides that explored participants' receptivity, information needs, and ideas for potential introduction of a generic 4-month DMPA-SC product. The method was described to respondents as being the same formulation as the existing DMPA-SC but with reinjection every 4 months with a 1-week “grace period” or reinjection window rather than a 4-week grace period for the 3-month product (Table 1).^26^ Interviewers also told participants that women using DMPA-SC every 4 months may have a quicker return to fertility and potentially fewer side effects due to the reduced medroxyprogesterone acetate accumulation associated with wider reinjection intervals. Respondents were asked about their perspectives on these injection characteristics as well as their views on self- versus provider administration and injection location on the body, including upper arm, abdomen, and thigh. In addition, respondents were presented with information about a 6-month injectable currently under development and asked about its potential acceptability among users and providers. The 6-month product was described as a subcutaneous injection delivered in a glass vial and syringe similar to existing DMPA-IM, not self-injectable, and with side effects similar to existing injectables. Respondents were asked how the new 4- and 6-month injectables may be received in Uganda and Nigeria and how multiple injections of varying durations would be received by users and potentially affect the market.

**TABLE 1.** Characteristics of Subcutaneous Injectable Products as Presented to Study Participants

	Existing 3-Month Product	4-Month Product Concept	6-Month Product Concept
Amount of medroxyprogesterone acetate	104 mg	104 mg	150 mg
Reinjection window	4 weeks	1 week	Not specified
Ability to self-inject	Yes	Yes	No
Return to fertility	Not described	Potentially shorter than existing 3-month	Not specified
Side effects	Not described	Potentially fewer than existing 3-month	Similar to existing 3-month
Regulatory approval/ state of evidence	Approved	Studies demonstrate effectiveness for 4-months	Effectiveness trial is ongoing

Data collection occurred July–November 2021 in Uganda and July 2021–February 2022 in Nigeria. IDIs and FGDs were conducted by the site investigators accompanied by a notetaker in either a private location convenient for the participant(s) or on a virtual platform (telephone or videoconference) and audio-recorded. IDIs and FGDs were conducted in English where possible, though some were conducted in Lugandan in Uganda and Yoruba in Nigeria.

All respondents gave their informed consent (oral in Nigeria and written in Uganda) and received a small stipend (approximately US$25) to cover opportunity costs associated with participating in the IDIs or FGDs.

### Ethical Approval

Ethical approval for this research was granted by the AIDS Support Organization Research Ethics Committee and Uganda National Council for Science and Technology in Uganda, the Lagos State University Teaching Hospital Health Research Ethics Committee in Nigeria, and FHI 360's Protection of Human Subjects Committee in the United States.

### Data Analysis

We used descriptive statistics to summarize the sociodemographic data and applied thematic analysis^27^ to analyze the qualitative data. For Uganda, all audio recordings were transcribed verbatim following a transcription protocol.^28^ IDIs and FGDs conducted in Lugandan were simultaneously transcribed and translated into English. Transcripts were imported into Nvivo 12 to facilitate data management. Then, 2 data analysts (DB and LR) independently applied codes for 1 transcript using an a priori coding structure based on the themes from the IDI/FGD guide. The analysts then met to assess intercoder agreement on the initial transcript, discuss discrepancies in code application, and revise the codebook, adding content codes as they emerged and refining codebook definitions. The data analysts conducted intercoder reliability checks until they were consistent in their code application, after which they independently coded the rest of the transcripts (the final set of themes and subthemes is included in the Supplement). Four analysts (2 who coded the transcripts above and another 2 who worked on analyzing the Nigeria data, CW and MV) then reviewed the coding reports to develop summaries for each code, including descriptions of subthemes, frequencies of responses, and illustrative quotes.

Data for Nigeria were analyzed separately from the Ugandan data. For Nigeria, 3 data analysts (CW, HA, and MV) listened to the audio recordings to complete a data extraction table that had themes that corresponded to the structure of the IDI/FGD guide and were similar to themes identified in the Ugandan data. Information from each recording was summarized and segmented by theme and added into the appropriate column, which also included illustrative quotes. The analysts then reviewed the information for each theme to complete summaries similar to what had been done for the Ugandan data.

At the final stage, we reviewed summaries for similar themes for both countries to compare the data. For this article, we narrowed our focus and are presenting summaries of the following 4 themes: (1) perspectives on the existing 3-month DMPA-SC product; (2) perspectives on the 4-month product concept; (3) perspectives on the 6-month product concept; and (4) preferred injectable characteristics. We also noted any difference in responses by respondent type (providers versus other FP stakeholders), and where none existed, responses were combined. While conducting data analysis, we assessed that we reached data saturation when no new themes and subthemes emerged and confirmed that no additional data collection was needed.

## RESULTS

We conducted 23 IDIs and 6 FGDs in Nigeria and 25 IDIs and 5 FGDs in Uganda, with a total of 92 participants. Participants were employed in a variety of organizations, mostly from NGOs, ministries of health, public and private sector health facilities, and community service organizations (Table 2).

**TABLE 2.** In-Depth Interview and Focus Group Discussion Participants by Country

	Nigeria, No. (%)	Uganda, No. (%)
In-depth interviews	(N=23)	(N=25)
Sex		
Male	9 (61)	12 (48)
Female	14 (39)	13 (52)
Organization type		
Nongovernmental organization/community service organization	9 (39)	16 (64)
Ministry of Health	4 (17)	5 (20)
University/academic institution	2 (9)	–
Private sector facility	6 (26)	1 (4)
Funder organization	2 (9)	3 (12)
Focus group discussions	(N=21)	(N=23)
Sex		
Male	3 (14)	8 (35)
Female	18 (86)	15 (65)
Organization type		
Nongovernmental organization/community service organization	5 (24)	2 (9)
Ministry of Health	1 (5)	4 (17)
Public health facility	11 (52)	12 (52)
Private sector facility	3 (14)	5 (22)
Funder organization	1 (5)	–

### Perspectives on the Existing 3-Month DMPA-SC Product

Interviews and FGDs began with a discussion of the existing 3-month DMPA-SC in the respective countries. All respondents had substantial familiarity with DMPA-SC, and many had been involved in recent introduction or scale-up activities for the product. In Nigeria, representatives from service delivery organizations said that the national roll-out of DMPA-SC is currently underway, but so far, it is not as popular as DMPA-IM. One respondent said that DMPA-SC is only used when DMPA-IM is not in stock. Others mentioned challenges with continuous supply of DMPA-SC. FP providers in Nigeria had differing opinions on the popularity of DMPA-SC, with a respondent saying that it is not as popular as other injectables. Other providers said that it is the first choice of many clients because it has fewer side effects, causes less pain at the injection site, and can be self-injected.

*It [DMPA-SC] is highly accepted. It has less side effects, very few people will tell you they are bleeding. Bleeding will reduce gradually. Because the needle is small, clients will choose it more and they are able to learn how to self-inject. They prefer to use it.* —Public health facility provider in Nigeria

In Uganda, service delivery partners, policymakers, and providers indicated that DMPA-SC is becoming more popular across the country, and a few said that it is the most preferred method among women. Reasons for increased use include reduced burden on health providers, ease of use, minimal side effects, clients' ability to conceal use from their partners, and reduced visits to the health facility.

Participants were also asked about preferences for provider versus self-administration among providers and users with the existing product. In Nigeria, all but 1 respondent said that provider administration was preferred because women were uncomfortable with or afraid of self-injection. Some Nigerian providers spoke about not teaching clients how to self-inject because they did not have time, did not feel confident that the client could self-inject on their own, or felt that clients would somehow abuse the product. In Uganda, provider administration was also identified as more popular than self-injection by most respondents. Many said that self-injection was still not widely available in the country, with few providers trained to support the practice. However, a minority of respondents noted that self-injection is becoming popular with some clients in areas where it has been promoted.

*Self-injection is more dominant; that is only in districts where women have been trained.* —NGO staff in Uganda

### Perspectives on a 4-Month DMPA-SC Product Concept

#### Perceived Benefits

After learning about the potential 4-month DMPA-SC from the interviewers, all respondents in Nigeria and almost all respondents in Uganda were in favor of integrating the new product into the method mix. In Nigeria, participants of all backgrounds thought the addition of the 4-month would give women more choices, which would, in turn, increase client satisfaction and expand the pool of new users. Others mentioned that the longer duration of effectiveness would be appealing for some women because it would reduce the frequency of clinic visits and, thus, travel time and costs of going to a facility. A Nigerian participant summarized the positive aspects of the 4-month injectable.

All respondents in Nigeria and almost all respondents in Uganda were in favor of integrating a new 4-month injectable product into the method mix.

*I have nothing against it if the 4 months is similar to the 3 months. That would mean less frequent injections and the longer it can last. I know it will be better for our clients as most will like to have something that they have to take less frequently but get more efficacy from it. So if it offers that and the undesirable effects are minimal and no different from the previous 1, then it will actually go a long way and demand will go up. It's something that even I will see as desirable to use.* —Nigerian MOH staff

In Uganda, respondents mentioned similar advantages, and a few also described a potential benefit to the supply chain, with governments needing to procure fewer doses in a year and women taking fewer doses home for self-injection.

*Given the reason of the supply challenges, instead of giving out 4 units they will be administering 3 and that will be cost effective for the country. And given the fact, DMPA-SC has taken toll in the country, there is a lot of high interest in it and given the fact that it is being used for self-injection there has been a concern by some of the districts and also the Ministry of Health that why should they give out 3 units for self-injection yet there are women who will be coming at the facility and it is not there, so, instead of 3 give out 2 assuming it is a 4-months injectable then it would really be good news. So, I feel good especially in terms of supplies which is a key challenge in the country.* —Public sector midwife in Uganda

#### Perceived Challenges

Reactions to a potential 4-month DMPA-SC were resoundingly positive, but participants in both countries identified some potential challenges with introducing the new method, including maintaining sufficient stock, counseling clients on a new method, possible higher cost, potential confusion among clients, and extra reporting burden, as it would be an additional FP method to report on. Some respondents also feared the 1-week reinjection window might not offer sufficient time for some women (especially those who travel or work) to come back for reinjection. Others mentioned that less educated women might have trouble returning on time. To address this, some suggested that more counseling and follow-up will be necessary to encourage clients to return on time.

*However, for less educated women, even for the 2- or 3-month injectable, some of them still miss their appointments. So for this new 4-month injectable, they may miss their appointment and will need intense follow-up from the provider in the form of reminder calls.* —Funder agency staff in Nigeria

A few Nigerian respondents also mentioned the reduced social and provider interaction with fewer facility visits as drawbacks of the method.

*If you talk to women, maybe at the grassroots, the opportunity to visit the hospital is welcome and can be seen as a social activity.* —NGO staff in Nigeria

#### Information and Support Needed for Introduction of a 4-Month DMPA-SC

When asked what additional information would be needed for the 4-month product to be adopted by their respective countries, respondents mentioned wanting more information on product effectiveness, potential side effects, differences between the new product and the 3-month DMPA-SC, and clarity on the reinjection window. Several also mentioned that training for providers would be needed. However, some said that training would be minimal unless there are major differences between the 3-month and 4-month products.

*If it is administered just like the 3-month, it won't take much. Just provide training to the provider and give them information about the new product. It won't take too much for it to be accepted, as long as we are confident that the information we are getting is reliable. They have to know that research has been done and that it's not just somebody bringing in another product to compete. If it's a different kind of product that is different from Sayana Press, information on how it should be administered needs to be provided.* —Private sector pharmacy staff in Nigeria

Other needs identified in both Nigeria and Uganda were the development of communication and counseling materials for clients, demand creation efforts, training of community distributors, and necessary changes to the supply chain.

*Definitely a change in quantification will have to come in and also a change in ordering, and also changes in procurement and planning, because remember you have been used to the 3-months dose and this time round you are having another dose that can go for 4 months. So, definitely those changes will come in at all levels right from the service delivery point for those who are able to quantify well, they will have to make those adjustments to the district as well as to the national center.* —MOH staff in Uganda

#### Potential for Off-Label Use of DMPA-SC as a 4-Month Method

Respondents were also asked about their potential support of a national-level policy change that would allow for off-label use of the currently available DMPA-SC for 4 months. Among policymakers in both countries, most said they would support a policy of off-label use if evidence of product safety and efficacy were strong. Those less supportive of a policy change cited concerns about product effectiveness, difficulty in implementing such a policy, and a belief that off-label use could be addressed by updating guidelines or just communicating the new usage of the method to providers. Those advocating for the latter based their views on the fact that both the 3- and 4-month products are inherently the same, and therefore, no changes are needed in the current policies.

*I think with regards to the MOH guideline, we don't need to revise the guidelines since it's the same product that is required that we will need to extend its period.* —MOH staff in Uganda

#### Preference for 4-Month Versus 3-Month DMPA-SC

Even though respondents in both countries almost unanimously approved of the introduction of the 4-month product, most did not indicate that providers would be more likely to recommend the 4-month over the 3-month. In Nigeria, several respondents focused on the importance of informed choice by contraceptive clients.

*You cannot choose for your client. What you do is to provide information to the client, and she will make a choice. It is against ethics for a provider to make the decision on which method the client should use. We can only counsel them on all the methods that are available and let them make an informed choice.* —Private sector midwife in Nigeria

In Uganda, several respondents also noted that method choice should be up to the client. However, several providers and policymakers thought that providers would prefer the 4-month over the 3-month product.

*Of course, me as a provider I would opt for the 4-month because I want to reduce the workload on my side but it's the client's choice.* —Public sector midwife in Uganda

Respondents in both settings pointed out that other factors would be important in the choice between the 2 methods among both providers and clients, including potential side effects, time to return to fertility, cost, and stock availability. Many respondents, especially providers, noted that if the 4-month had a quicker return to fertility (though this was not further defined), it would be more attractive to clients and providers.

Respondents in both settings noted certain factors would be important in the choice between the 3-month and 4-month methods among both providers and clients, including potential side effects, time to return to fertility, cost, and stock availability.

*Obviously it is good, because by the time a woman stops she wants to return to fertility. Actually 1 of the myths and misconceptions that we are fighting is prolonged return to fertility. Some women in the villages are not taking contraceptives because they believe once you take contraceptives all the eggs will be killed and you will never even get pregnant again, isn't it?… So, for me this 1 plays exactly into what we are trying to address, the myths and misconceptions.* —Community service organization staff in Uganda

Respondents were also asked how reactions to the product may differ depending on how it was presented: either as a 3-month method with a 4-week reinjection window or a 4-month method with a 1-week reinjection window. In Nigeria, several respondents said they preferred the former.

*The 1 with the longer window period is more preferable to me; some people may not be able to return to clinic quick enough, they can feel safe in the fact that there is a window period within which they can come in for the injections.* —Private sector nurse in Nigeria

Several others felt that it did not make a difference and how the method was presented could be case dependent.

*There's no 1 answer to this question. It will suit different circumstances….I don't see any advantage or disadvantage. I don't see 1 answer that will fit all.* —NGO staff in Nigeria

In Uganda, responses were similarly mixed, though more said that they thought the 4-month option with a 1-week reinjection window would be best because it would entail fewer injections and save resources. Some mentioned that it would be easier to counsel on a 1-week reinjection window and the sense of urgency would encourage users to return for reinjection.

*We calculate for them and we give them the actual date they are coming back because if you don't calculate for them, that will be mismanaged.* —Public sector nurse in Uganda

As in Nigeria, several Ugandan respondents said that the framing of the period of effectiveness and reinjection window could be case dependent and that there is no real difference between the 2 options.

*Honestly, in terms of efficacy, I mean, what is it? What is better? I don't know. For me, I'm looking at the operations point of view in the system. And like I said before, I don't think there is, in my opinion, a significant difference.* —Funder agency staff in Uganda

### Perspectives on a 6-Month Injectable Product Concept

In Uganda, all respondents said they thought a 6-month injectable would fit well into their health system and cited advantages, such as less frequent health facility visits, reduced burden on providers, and potentially simplified product procurement logistics and storage.

*Definitely there are a number of advantages related to … logistics to handle the product. The more quantity you get the more you have to spend in terms of logistics to handle. And this handling we are looking at the supply chain right away the storage you need if you do only 1 vial instead of keeping let's say 2, so that means it is a reduction.* —NGO staff in Uganda

Only a couple of respondents in Uganda mentioned potential downsides to a 6-month injectable, including potential confusion among users if there are multiple injectables available and potential longer experience of side effects.

*The only thing I am worried of are the side effects. If they are going to last longer then it is going to be a problem. Because already women are complaining about the side effects but if this registers a longer experience of side effects then to me that's a huge negative. And remember, in Africa, men [say] 1 of the reasons they don't like their women to take family planning is because of having longer bleeding periods. So, if you are going to have a product that is going to bring a worse bleeding period then we shall end up impacting negatively on our family planning program. On the other hand, yet I would like to have a 6-months [product] but if the side effects are longer then I don't see the benefits.* —Community service organization staff in Uganda

In Nigeria, almost all respondents also thought that a 6-month injectable would be welcomed by users and that the additional option would be good for expanding access to contraception. However, a few also identified potential downsides to a 6-month product, including that it would not be self-injectable, that some women may not remember to go for reinjection, and that if the product had side effects, they would last for a 6-month period. A few respondents had mixed feelings about the 6-month product, identifying both positive and potentially negative consequences.

*Side effects matter. Six months is long [to endure]. I'm not really comfortable about that. One positive side is that there are less shots. Women will forget, unless you are calling them to remind them.* —University/hospital staff in Nigeria

Although some respondents expressed concern about side effects associated with the 6-month product, most thought that they could be dealt with through appropriate provider counseling and available treatments.

*Side effects can be managed; they [clients] can be told what to do when they exhibit side effects. There are things they can do to manage them.* —NGO staff in Nigeria

When asked whether they thought existing 3-month injectable users would switch to the 6-month, almost all respondents in both countries said they thought many would. In Uganda, some participants figured that the switch may not happen immediately, saying that women will wait for others to switch first and hear their experiences.

*Given the advantages of the 6 months product that we will be having over these other products but at the same time we have to keep it in mind that there are those ones who will switch after having heard their other colleagues giving testimonies on this other product, so, it may not take very fast but later on, it can be picked after getting testimonies from those who have already used the product of the 6 months.* —MOH staff in Uganda

In Nigeria, respondents gave wide-ranging estimates of the proportion of injectable users they figured may switch to a 6-month product. Several respondents described types of users they assume may switch, such as those who have infrequent sex, younger women who are “more adventurous” and open to trying new things, working women who would welcome fewer clinic visits, and women who have completed their family size.

### Preferred Injectable Characteristics

Respondents also shared their perspectives on general injectable product characteristics, including optimal duration of effectiveness, the importance of self-injection, and preferred injection site.

#### Preferred Duration

In Uganda, most respondents said that a 6-month product would be the best duration. Two respondents (both providers) said that they preferred 4 months, and other responses included 3 months, 4–6 months, and 1 year. In Nigeria, respondents identified a range for the ideal duration, with the majority preferring 3–6 months, followed by 6–12 months, and 1 year or more. Reasons for preferring a 6-month product in Uganda were similar to the advantages of a 4-month product described earlier, including fewer clinic visits, cost savings for clients, and reduced workload for providers. Among the few who said that a shorter-acting injectable would be better, concern over prolonged side effects was cited as the reason for their preference. One Ugandan provider said that her preference would depend on whether she was working in the public or private sector. If the former, she would prefer 6 months to reduce workload, but if she were working in the private sector, she would prefer a shorter duration to bring in more revenue. A couple of respondents in both countries said that an ideal duration does not exist because users have different preferences and needs, so more options would be better.

*I don't think anybody can tell any ideal, with how many options are existing and many more options will exist, because some people want a 10-year IUD, some people want a 1-month injectable, some people want an emergency contraception, so I don't think you can say there is an ideal. I think if there are many more options available to women, the better. And every woman has a different point and reproductive life cycle and preferences for childbearing, so it depends on what the woman is looking for.* —NGO staff in Uganda

#### Preference for Provider- Versus Self-Injection

Respondents in Uganda were generally more supportive of a self-injectable product than respondents in Nigeria. Some respondents in Nigeria pointed out that contact with a health provider was particularly important for a longer-acting method (e.g., like a 6-month injectable) for both reminding users to get their injection and to check in on side effects and other needs.

*If it's a longer-acting method, the higher the risk of forgetting to take it when due. There can be a way of prompting women or giving reminders. A hospital visit might be a better prompt than reminding oneself to take an injection. A return visit is more likely to be remembered; sometimes when things are in our control we procrastinate. But booking an appointment and scheduling a visit might help to prompt women to visit.* —NGO staff in Nigeria

In Uganda, several participants viewed the potential for self-injection as very important, particularly for busy women and those who may be using discreetly. One respondent brought up the importance of advancing self-care options for increasing FP access and use. However, several other Ugandan respondents expressed sentiments similar to those in Nigeria that users would benefit from contact with a health provider with a less frequently administered method. Others mentioned that self-injection is not that popular with existing Sayana Press users and that injectables have been provider administered for a long time, so people are used to this.

#### Preference for Injection Location

The 6-month injectable described in this research would require administration in the abdomen or thigh. Respondents were asked whether these locations would be less acceptable than injection in the arm or elsewhere. Most Nigerian respondents did not think that they would be, and some mentioned that other injections are given in these locations (abdomen and thigh). However, a few noted that some women would not want to expose their thigh or abdomen to a male provider, so the preferred injection site could depend on whether the product was self- or provider-administered.

*It would depend if it were SI [self-injection] or provider-based. More religious women would not be injected by a man in the thigh or abdomen and the arm is much more acceptable culturally.* —NGO staff in Nigeria

In Uganda, responses varied. A few respondents said that injection site preference would depend on the individual and that some women would not want to expose their thigh or abdomen to a male provider. A few others said that site did not matter and that quality of the service provision and effectiveness of the product were more important. Other Uganda respondents mentioned the importance of a private location for injection and that more research should be done to understand user preferences for injection site.

## DISCUSSION

This research explored the perspectives of FP providers, policymakers, program implementers, and other stakeholders on the potential introduction of new contraceptive injectables lasting 4 and 6 months in Uganda and Nigeria. Both new product concepts were received positively for their potential to grow the contraceptive methods mix and expand choice; however, respondents' views on the new products were nuanced. Overall, participants liked that the new injectables offered longer pregnancy protection, reduced clinic visits for users and, thus, workloads for providers, and potential cost savings for commodity procurement. However, their positive perspectives assumed that the new products would not have worse side effect profiles than the existing 3-month DMPA-SC currently being scaled up in both countries.

We conducted this study in parallel with market research focused on potential user attitudes toward the new injectables, which included similar questions.^16^ Many of the sentiments expressed by FP stakeholders about both the 4- and the 6-month injectables were consistent with those of potential users. Stakeholders and users in both settings identified a benefit in reduced frequency of facility visits, expressed preference for provider-administered injectables, and raised concern about side effects, particularly for a 6-month injectable. Nigerian stakeholders and potential users both mentioned reduced provider interaction as a potential concern with a longer-duration injectable—a theme not mentioned by either group in Uganda. Regarding the reinjection window, though viewpoints were mixed, more stakeholders and potential users in Uganda thought the 4-month option with a 1-week reinjection window would be better, whereas more Nigerian stakeholders and potential users preferred the 3-month with longer reinjection window. Across all groups, the 6-month injectable was viewed as easier to distinguish from existing injectables compared to the 4-month. Although several nonprovider stakeholders in both countries expressed concern about confusion between 3-month and 4-month injectables, providers and potential users^16^ in both countries did not feel that confusion between products would be an issue and welcomed an expansion of the number of injectable options in their country.

Stakeholders and users in both settings identified a benefit in reduced frequency of facility visits, expressed preference for provider-administered injectables, and raised concern about side effects, particularly for a 6-month injectable.

Although stakeholder respondents and potential users in the market research recognized advantages of both the 4- and 6-month injectables, the vast majority thought that the longer-duration injectable offered the most promise. Even without the option of self-injection, respondents felt that the 6-month injectable would be appealing to users and providers, some mentioning that self-injection of the existing 3-month DMPA-SC is not widely practiced. Although responses indicated that self-injection is more prevalent in Uganda than in Nigeria, the sentiment that self-injection has not been taken up extensively in either country aligns with recent Performance Monitoring and Action survey data from 4 sub-Saharan African settings, including Lagos and Kano states in Nigeria. These data show that only up to 17% of current DMPA-SC users across the settings report self-injecting and that among contraceptive nonusers considering use of injectables in the future, less than 12% wished to self-inject.^29^ The lack of enthusiasm for self-injection both in the present research as well as the Performance Monitoring and Action survey results could be the result of a number of factors, including lack of awareness due to limited marketing campaigns, insufficient provider training and promotion among health workers, DMPA-SC supply challenges that discourage providers from prescribing and supporting self-injection, among other causes.^29^ Fear of self-injection has also been identified as a barrier to self-injection,^30^ and this also came up several times in the present study.

This research included voices of key FP stakeholders ranging from service providers at different levels in both the public and private sectors, NGO program managers, MOH officials, funders, and others. We were fortunate to be able to interview participants of the national-level FP working groups in both Nigeria and Uganda, ensuring that opinions of key decision-makers were included. The research also benefited from a concurrent market research effort, which collected perspectives from potential users. Site investigators were involved in both research efforts and were able to triangulate both lines of inquiry and responses.

### Limitations

Our research does suffer from some limitations, however, including that most interviews were conducted with stakeholders in urban or peri-urban areas. Although we recruited respondents from areas outside the capital cities in both countries, our data may not fully reflect perspectives from more rural areas. Also, we interviewed national-level stakeholders, but our sample is not nationally representative, and we do not claim that the results reflect the views and opinions of all FP stakeholders and providers in the 2 countries. Despite these limitations, we found several consistent themes within and across sites and believe that our findings provide meaningful insights into the opportunities and potential challenges for introducing new injectables lasting 4 and 6 months.

## CONCLUSION

New injectables offer the potential to meet the needs of more users. Although we found mixed opinions on the benefits and potential drawbacks of introducing a 4-month DMPA-SC injectable alongside the existing 3-month option in Uganda and Nigeria, almost all agree that expanding the method mix will improve access and use of FP. Engaging key stakeholders from across the health sector will be key to the successful introduction of any new injectable that reaches the market.

## Supplementary Material

23-00214-Callahan-Supplement.pdf

## Notes

**Peer Reviewed**

**First published online:** December 6, 2023.
