# Supplementary material for: Service Delivery Considerations for Introducing New Injectable Contraceptives Lasting 4 and 6 Months in Nigeria and Uganda: A Qualitative Study
Source: Glob Health Sci Pract. 2023 Dec 22;11(6):e2300214. doi: 10.9745/GHSP-D-23-00214 (PMC10749649; doi:10.9745/GHSP-D-23-00214)
Supplement: 23-00214-Callahan-Supplement.pdf [file 23-00214-Callahan-Supplement.pdf]

Supplement to: Callahan RL, Burke HM, Lawton A, et al. Service delivery considerations for introducing new injectable contraceptives lasting 4 and 6 months in Nigeria and Uganda: a qualitative study. *Glob Health Sci Pract*. 2023;11(6):2300214. https://doi.org/10.9745/GHSP-D-23-00214

Supplement

List of themes and sub-themes

- **DMPA-SC Knowledge, current use and guidance followed**
  - DMPA-SC knowledge/use
  - Guidance followed

- **Acceptability of 4-month product and changes needed at policy-level**
  - Reaction to 4-month product introduction
    - Additional information needs
    - Trusted sources of information
  - Steps need to motivate change in national policy
  - Impact of 4-month product on guidelines

- **Impact of the 4-month product at health system- and provider-levels**
  - Changes needed at:
    - Provider-level
    - Health system-level
  - Challenges/Barriers to provision
  - Impact of intro on training & supervision
    - Challenges & solutions
  - Other provider-level communications needed
  - Systemic changes needed for Introducing the product
    - Specific mechanisms
    - Impact on clinic volume, data & monitoring, logistics and distribution
  - Role of cost in provision
    - Incentives
    - Disincentives
  - Ensuring quality assurance

- **Acceptability of 4-month product among providers and administration**
  - Provider preference: 3 or 4 month product?
  - Impact of potentially:
    - Reduced side effects
    - Quicker return to fertility

- **Acceptability of 4-month product among clients**
  - Perceived acceptability among potential clients
    - Benefits & drawbacks
  - Anticipated client questions/concerns
  - Impact of potentially reduced side effects on client preference
  - Impact of potentially quicker return to fertility on client preference
  - "Ideal" Target Clients

  - Client preference on the impact of longer intervals on client confidence & recall
  - Client preference for 'grace period': 1) 3 months + 4 weeks *OR* 2) 4 months + 1 week

- **Acceptability of 6 month product**
  - Acceptability of 6-month product with providers
    - Potential positives and negatives
  - Acceptability of 6-month product with clients
    - Potential positives and negatives
  - Concern for inability to remove/reverse product for 6 months
  - Likelihood of switching to 6-month product
    - Demand forecast

- **Optimal length, ranked attributes, and optimal # of injectables**
  - Optimal injectable duration - provider
    - Ranked importance of:
      - Administration type
      - Location of injection
      - Availability (e.g., clinic-only)
  - Optimal injectable duration - client
    - Ranked importance of:
      - Administration type
      - Location of injection
      - Availability (e.g., clinic-only)
  - Optimal # of injectables in the Market
    - [Private sector] Impact on pricing & sales

- **Differentiating the methods and mitigating confusion**
  - Impact of different durations on market
    - Potential provider confusion + solutions
    - Issues with forecasting, stocking + solutions
  - Impact of different durations on market
    - Potential client confusion + solutions
  - Preventing confusion with multiple durations
    - 3 & 6-month vs 3,4 & 6-month
  - Recommendations for promoting/differentiating 4-month product
  - Recommendations for promoting/differentiating 6-month product

- **Insurmountable Challenges**
  - Suggested Mitigations
